# Double-sided endoscopic submucosal dissection with two scopes and adaptive traction for an ileocecal neoplasia: when ileostomy allows simultaneous dissection on the two edges

**DOI:** 10.1055/a-2523-2523

**Published:** 2025-02-11

**Authors:** Aïmène Khiari, Pierre Lafeuille, Clara Yzet, Florian Rostain, Alexandru Lupu, Jérôme Rivory, Mathieu Pioche

**Affiliations:** 1 Gastroenterology and Endoscopy Unit, Edouard Herriot Hospital, Hospices Civils de Lyon, Lyon, France; 2 Amiens University Hospital, Université de Picardie, Amiens, France

Endoscopic submucosal dissection (ESD) is a standard treatment for superficial colorectal neoplasia. Size greater than 40 mm and right colon location present significant challenges 1 2 . The ileocecal valve (ICV) is considered one of the most difficult locations. Nevertheless, it has been shown that ESD in this area is both safe and effective, especially when using traction devices 3 . A recent French study of 106 patients demonstrated that traction-assisted ESD for ICV lesions is a safe and feasible option 4 . Factors associated with non-R0 resection include lesions covering ≥75% of the ICV, involving the anal lip, or spreading across more than two sites on the ICV 4 .

We report the case of a 70-year-old patient who presented with bowel obstruction due to an abscessed sigmoid adenocarcinoma. An initial colostomy allowed for a colonoscopy, which revealed an 8-cm heterogeneous granular laterally spreading tumor in the cecum invading the anal lip of the ICV. The patient subsequently underwent sigmoid resection and anterior rectal resection with protective ileostomy.

Owing to the size of the lesion and ICV involvement, we decided to perform ESD using a two-scopes technique ( Video 1 ). We used an Olympus PCF 190L (Olympus, Tokyo, Japan) colonoscope through the anus and an Olympus PCF 190Ti colonoscope through the ileostomy ( Fig. 1 ). Dissection was performed in 240 minutes using a DualKnife (Olympus) connected to an Erbe VIO3 electrosurgical unit (Erbe Elektromedizin GmbH, Tübingen, Germany) for each scope, and using adaptive traction (A-TRACT; ATRACT Device and Co., Lyon, France) 5 . The specimen was a 10 × 9 cm adenoma with focal high grade intraepithelial neoplasia, resected en bloc with clear margins.

**Fig. 1:**
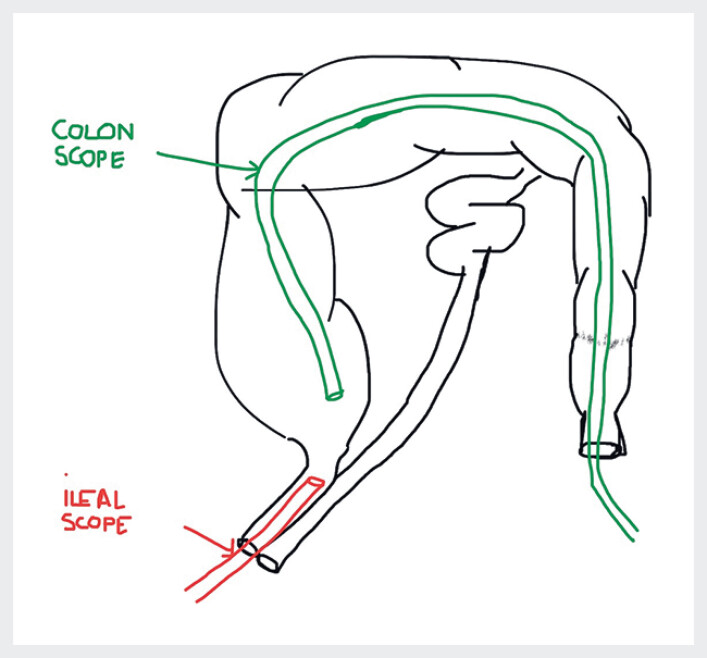
Schematic representation of the two-scope endoscopic submucosal dissection technique: one scope via the colon and the other via the ileum.

Double-sided endoscopic submucosal dissection with two scopes and adaptive traction for an ileocecal neoplasia.Video 1

The patient had no immediate or delayed complications such as perforation, bleeding, or stricture. Follow-up surgery was performed to restore bowel continuity and was also free of complications.

This case highlights the feasibility and safety of ESD using a two-scope technique for large ICV lesions in patients with stoma.

Endoscopy_UCTN_Code_TTT_1AQ_2AD

## Notes

Endoscopy E-Videos https://eref.thieme.de/e-videos
*E-Videos* is an open access online section of the journal *Endoscopy* , reporting on interesting cases and new techniques in gastroenterological endoscopy. All papers include a high-quality video and are published with a Creative Commons CC-BY license. Endoscopy E-Videos qualify for HINARI discounts and waivers and eligibility is automatically checked during the submission process. We grant 100% waivers to articles whose corresponding authors are based in Group A countries and 50% waivers to those who are based in Group B countries as classified by Research4Life (see: https://www.research4life.org/access/eligibility/ ). This section has its own submission website at https://mc.manuscriptcentral.com/e-videos .
